# Dataset of fire tests with lithium-ion battery electric vehicles in road tunnels

**DOI:** 10.1016/j.dib.2022.108839

**Published:** 2022-12-16

**Authors:** Peter Sturm, Patrik Fößleitner, Daniel Fruhwirt, Simon Franz Heindl, Oliver Heger, Robert Galler, Robert Wenighofer, Stefan Krausbar

**Affiliations:** a Graz University of Technology, Institute of Thermodynamics and Sustainable Propulsion Systems, Austria; b Graz University of Technology, Institute of Vehicle Safety, Austria; c ILF Consulting Engineers Austria GmbH, Austria; d Montanuniversität Leoben, Chair of Subsurface Engineering, Austria; e Austrian Fire brigade Association, Austria

**Keywords:** Battery electric vehicles, Car fire, Heat release rate, Fire-fighting, Underground systems, Data set, BEV, Battery electric vehicle, CFD, Computational fluid dynamics, HRR, Heat release reate

## Abstract

A set of five fire tests involving battery electric vehicles and conventional cars was performed in a tunnel. This data article provides the dataset of some of the determined parameters: air temperature, air velocity and heat release rate. The air temperature was measured at several locations and at different heights, distributed in the cross section of the tunnel. The velocity of the incoming air was also measured. The third parameter, the heat release rate (HRR), was calculated based on the enthalpy flow before and after the fire location. This parameter is important for characterizing the size of a vehicle fire.

The data provide a reference for the evaluation of BEV fires and could be taken as reference for further fire studies. They might be also of interest to research groups dealing with simulation applications, like three-dimensional CFD simulations.

**Specifications Table**

**Table:** 

Subject	Engineering
Specific subject area	Thermodynamics and experimental fire tests in road tunnels
Type of data	Table Graph
How the data were acquired	**Temperature** ­ Measuring technique: thermocouples type K, class1 ­ Unit:°C **Velocity** ­ Measurement technique: distance-averaged ultrasonic transit time difference measurement ­ Unit: m/s **Heat release rate (HRR)** ­ Calculated using the temperature measurements of the enthalpy flow before and after the fire location. ­ Unit: MW
Data format	Analyzed Filtered
Description of data collection	Temperature data are collected by thermocouples, distributed across the cross-section at various points. Velocity data was collected by a distance-averaged ultrasonic transit time difference measurement.
Data source location	Institution: Graz University of Technology City/Town/Region: Styria/ Country: Austria
Data accessibility	Repository name: Repository of Graz University of Technology Data identification number: 10.3217/ysgwn-3a318 Direct URL to data: 10.3217/ysgwn-3a318
Related research article	P. Sturm, P. Fößleitner, D. Fruhwirt, R. Galler, R. Wenighofer, S.F. Heindl, S. Krausbar, O. Heger, Fire tests with lithium-ion battery electric vehicles in road tunnels, Fire Safety Journal 134 (2022) 103695. 10.1016/j.firesaf.2022.103695.

## Value of the Data

• The data in this article [2] are useful because relevant test results from real-scale vehicle fire tests in a road tunnel are provided. This concerns tests of vehicles with lithium-ion batteries as well as vehicles powered by an internal combustion engine.
• The data are of interest to research groups and to all those who are interested in the effects of vehicle fires. In the presented experiments, the difference of vehicle fires of battery-electric passenger cars compared to conventional vehicles (internal combustion engines) in a road tunnel was investigated.
• Further uses of the data represent an added value, especially for simulation applications such as three-dimensional CFD simulations. On the other hand, they also provide a reference if it is planned that further fire studies will be carried out on such a large scale. In addition, the data can also be used for model fire tests.

## 1 Introduction

In the last years, concerns were voiced with respect to the fire safety of vehicles powered by new energy carriers, primarily battery electric vehicles. This resulted in a series of experiments, commissioned by the Austrian Government concerning the effect of incidents with battery electric vehicles on tunnel safety. In a series of 5 fire tests, the air temperature in the tunnel, the air velocity and the heat release rate of the burning vehicle has been evaluated. The objective of this project was to compare the burning behaviour of vehicles with different energy storage technologies: battery electric vehicles and conventional vehicles (powered by diesel or gasoline). These data [2] allow a detailed insight into the graphs and tables presented in the original research article [1].

## 2 Data Description

All tests were carried out in a tunnel, which is quite similar to the horseshoe profile of a road tunnel. The tunnel height measures about 7.5 m, the cross-sectional area is 52 m² and the perimeter is about 26.5 m.

The sensors were distributed quite evenly across the cross-section at various points of the tunnel. Eight temperature sensors were at the soffit directly above the fire at position 1. Table 1 shows the coordinates, where the x-axis corresponds to the longitudinal axis of the tunnel, the z-axis stretches from the road surface to the tunnel ceiling, transverse to this is the y-axis. The origin of the coordinate system is located at the fire scene resp. the vehicle.

**Table 1:** Location of sensors at position 1

Sensor	x	y	z
T 1.1	-5.25 m	0 m	7.5 m
T 1.2	-3.75 m	0 m	7.5 m
T 1.3	-2.25 m	0 m	7.5 m
T 1.4	-0.75 m	0 m	7.5 m
T 1.5	0.75 m	0 m	7.5 m
T 1.6	2.25 m	0 m	7.5 m
T 1.7	3.75 m	0 m	7.5 m
T 1.8	5.25 m	0 m	7.5 m

Regarding to Table 2, eight sensors were positioned 16 m after the burning vehicle at position 2.

**Table 2:** Location of sensors at position 2

Sensor	x	y	z
T 2.1	16 m	0 m	7.5 m
T 2.2	16 m	0 m	7.0 m
T 2.3	16 m	0 m	6.5 m
T 2.4	16 m	0 m	6.0 m
T 2.5	16 m	0 m	5.0 m
T 2.6	16 m	0 m	4.0 m
T 2.7	16 m	0 m	3.0 m
T 2.8	16 m	0 m	2.0 m

Another 16 sensors were in a distance of 32 m at position 3 (Table 3). Besides the eight centered sensors, four sensors each were mounted on the left and right side.

**Table 3:** Location of sensors at position 3

Sensor	x	y	z
T 3.1	32 m	0 m	7.5 m
T 3.2	32 m	0 m	7.0 m
T 3.3	32 m	0 m	6.5 m
T 3.4	32 m	0 m	6.0 m
T 3.5	32 m	0 m	5.0 m
T 3.6	32 m	0 m	4.0 m
T 3.7	32 m	0 m	3.0 m
T 3.8	32 m	0 m	2.0 m
T 3.9	32 m	2.5 m	6.0 m
T 3.10	32 m	2.5 m	5.0 m
T 3.11	32 m	2.5 m	4.0 m
T 3.12	32 m	2.5 m	3.0 m
T 3.13	32 m	-2.5 m	6.0 m
T 3.14	32 m	-2.5 m	5.0 m
T 3.15	32 m	-2.5 m	4.0 m
T 3.16	32 m	-2.5 m	3.0 m

In order to measure the temperature of the incoming air, one temperature sensor was placed 30 m upstream to the fire (position 0). Furthermore, the air velocity was also measured there, see Table 4.

**Table 4:** Location of sensors at position 0

Sensor	x	y	z
T QS	-30 m	-	-
Velocity	-30 m	-	-

In total, 5 fire tests with passenger cars were carried out [1], specifically 3 battery electric vehicles as well as 2 vehicles powered with an internal combustion engine. Further information can be found in Table 5.

**Table 5:** Overview of tested vehicles

Test	Vehicle type	Propulsion type	Model year
BV01	Compact car	Battery electric vehicle	2020
BV02	Utility van	Battery electric vehicle	2016
BV03	SUV	Internal combustion engine	2020
BV04	Utility van	Internal combustion engine	2010
BV05	SUV	Battery electric vehicle	2020

The measurement data of each test are saved in a separate file and can be obtained from [2]. Each file consists of three worksheets, including the data of temperature, velocity and heat release rate.

• Worksheet ``**Temperature**'': the measured air temperature with units of (°C) in 5s intervals.
• Worksheet ``**Velocity**'': the measured air velocity at the side of the inflow to the fire scene is given in (m/s) in 5s intervals.
• Worksheet ``**HRR**'': the calculated heat release rate in unit of (MW) in 5s intervals.

## 3 Experimental Design, Materials and Methods

**Temperature:** Temperature measurements were performed using type K, class 1 sensors. Directly above the fire site, 8 temperature sensors were mounted along the tunnel ridges. In addition, temperature cords were attached centrally at a distance of 16 m and 32 m downstream of the fire location, which recorded a vertical profile of the temperature at 0.5 meter to one meter intervals from ridge to ground. Four additional temperature sensors each were attached to the left and right of center 32 m downstream of the fire location.

**Velocity:** Velocity measurements were performed using a distance-averaged ultrasonic transit time difference measurement, DURAG D-FL. The measurement of the flow velocity of the inflow to the fire location was carried out 30 m upstream of the fire location.

**HRR (heat release rate):** The heat release rate was calculated using the temperature measurements of the enthalpy flow before and after the fire location. The difference was used to determine the effective amount of heat released into the air stream.

$$HRR={\dot{H}}_{out}-{\dot{H}}_{in}={\dot{m}}_{out}*c_{p}*\left(T_{out}-T_{0}\right)-{\dot{m}}_{in}*c_{p}*\left(T_{in}-T_{0}\right)$$

However, the values are subject to some uncertainty due to the uncertainties in calculating the enthalpy flux downstream of the fire location (${\dot{H}}_{out}$). The data of the HRR is much more of an estimation.

## Ethics Statements

The data does not involve human subjects, animal experiments and was not collected from social media platforms.

## CRediT Author Statement

**Peter Sturm:** Funding acquisition, Writing – review & editing, Methodology, Supervision; **Patrik Fößleitner:** Writing – original draft, Investigation, Methodology, Measurements; **Daniel Fruhwirt:** Investigation, Methodology, Measurements; **Simon Franz Heindl:** Investigation; **Oliver Heger:** Investigation, Methodology; **Robert Galler:** Investigation, On-site Supervision; **Robert Wenighofer:** Investigation; **Stefan Krausbar:** Investigation.

## Declaration of Competing Interest

The authors declare that they have no known competing financial interests or personal relationships that could have appeared to influence the work reported in this paper.

## Data Availability

"BRAFA" - Brandauswirkungen von Fahrzeugen mit alternativen Fahrzeugantrieben, Teil 1 (Original data) (Repository of Graz University of Technology).
